# Supplementary material for: A hotspot phosphorylation site on SHP2 drives oncoprotein activation and drug resistance
Source: Nat Commun. 2026 Mar 3;17:3383. doi: 10.1038/s41467-026-70060-8 (PMC13066003; doi:10.1038/s41467-026-70060-8)
Supplement: Supplementary file 1 — Supplementary Information [file 41467_2026_70060_MOESM1_ESM.pdf]

Supplementary Table 1: Crystallographic data collection and refinement statistics

PDB entry	9R16

Data processing

Space group	$P2_1$
Unit cell dimensions<br>a, b, c / Å<br>α, β, γ / °	 45.17, 212.16, 54.92<br>90.0 96.5 90.0
Resolution[a]	34.3-2.63 (3.04-2.63)
Number of reflections<br>Total<br>Unique	 144690 (14234)<br>21059 (2106)
R_{meas}	0.266 (0.834)
R_{pim}	0.101 (0.317)
Mean I/σI	5.7 (2.5)
$CC_{1/2}$	0.987 (0.718)
Multiplicity	6.9 (6.8)
Completeness<br>Spherical<br>Ellipsoidal	 69.1 (19.5)<br>89.0 (51.1)
Wilson B-factor / $Å^2$	54.6
Refinement	
Resolution	27.8-2.63 (2.82-2.63)
R_{work}	0.280 (0.305)
R_{free}	0.304 (0.333)
Number of atoms	8499
Average B-factor	51.0
R.M.S. deviations<br>Bond lengths / Å<br>Bond angles / °	 0.004<br>0.62
Ramachandran plot / %<br>Favored<br>Allowed<br>Outlier	 97.6<br>2.2<br>0.2
Clashscore	0.49

Supplementary Table 2: HDX/MS experiments summary

Dataset	**SHP2 Rep1**	**SHP2 Rep2**	**SHP2 Rep3**
Protein states	WT and Y62D	WT and Y542D_Y580D	Y62D and Y542D_Y580D
Date	10/02/2023	02/23/2024	01/14/2025
Protease column	Fungal protease XIII/pepsin	Fungal protease XIII/pepsin	ANPEP/pepsin
Reaction details	25mM Tris-HCl, 150mM NaCl, 2mM DTT, temperature 15 °C, final D_2O concentration 90%		
Time course (s)	0, 30, 120, 480, 960, 1920, 3840, 7680	0, 30, 120, 480, 960, 1920, 3840, 7680	0, 30, 100, 480, 1920, 7680, 40000
# of time points	7	7	6
# of peptides	213	333	267
Sequence coverage	78.9%	87.9%	90.5%
Average peptide length / Redundancy	10.3 length, 3.6 redundancy	12.6 length, 6.9 redundancy	10.2 length, 4.5 redundancy

Supplementary Table 3: gRNA and Primer seuences used in this study. gRNAs were purchased from GeneScript. Primers were synthesized and obtained from IDT.

	Sequences
PTPN11_gRNA1	GATTACTATGACCTGTATGG
PTPN11_gRNA2	GCGCACTGGTGATGACAAAG
PTPN11_gRNA3	TTACTATGACCTGTATGGAG
Y62D_Forward	CACTGGTGATgatTATGACCTGTATG
Y62D_Reverse	TTCTGAATCTTGATGTGG
Y62F_Forward	CACTGGTGATttcTATGACCTGTATG
Y62F_Reverse	TTCTGAATCTTGATGTGGG
Y542D_Forward	AGGGCACGAAgatACAAATATTAAG
Y542D_Reverse	TTCTGAATCTTGATGTGGG
Y542F_Forward	AGGGCACGAAttcACAAATATTAAG
Y542F_Reverse	TTCCTCTTGCTTTTCTGC
Y580D_Forward	TGCTAGAGTCgatGAAAACGTGG
Y580D_Reverse	CTGTCTTCTCTCATTTCTGC
Y580F_Forward	TGCTAGAGTCttcGAAAACGTGG
Y580F_Reverse	CTGTCTTCTCTCATTTCTG
pLX304_Forward	AACAGCAGAAAAGTTTCAGAGGTAAGCCTATCCCTAACCCTCT
pLX304_Reverse	AACCATCTCCGCGATGTCATTGATCCCGACAGTTAGCCAG
SHP2_Ins_pHAT_Forward	tcatcaccatcaccatcacaacactagtagcgctaccatgATGACATCGCGGAGATGGTT
SHP2_Ins_pHAT_Reverse	gatttaggtgacactatagaatactcaagcttatgcatgcTCATCTGAAACTTTTCTGCTGTTGC
SHP2_Ins_pLX304_Forward	CTGGCTAACTGTCGGGATCAATGACATCGCGGAGATGGTT
SHP2_Ins_pLX304_Reverse	CTGGCTAACTGTCGGGATCAATGACATCGCGGAGATGGTT
pGEX_sequencing_Forward	TGGTAGAACGAAGCGGCG
pGEX_sequencing_Reverse	CGACACCACCACGCTGG
His-tag_Forward	caccaccacGGAATTCCGGGCGGGAGG
His-tag_Reverse	atgatgatgCATGAATACTGTTTCCTGTGTGAAATTGTTATCC

Supplementary Figures:

a

Conservation of SHP2 Y62 across animal species

Species	Common name	Sequence	Bolded residue
H. sapiens	*Human*	KIQNTGD**Y**YDLYGGE	Y62
P. troglodytes	*Chimpanzee*	KIQNTGD**Y**YDLYGGE	Y61
M. musculus	*House mouse*	KIQNTGD**Y**YDLYGGE	Y62
M. domestica	*Housefly*	KIQNTGD**Y**YDLYGGE	Y62
G. gallus	*Redfowl*	KIQNTGD**Y**YDLYGGE	Y62
A. carolinensis	*Green Anole*	MIRCQDM**K**YDVGGGE	K62
X. tropicalis	*Western Clawed Frog*	KIQNTGD**Y**YDLYGGE	Y62
D. rerio	*Zebrafish*	KIQNTGD**Y**YDLYGGE	Y62
D. melanogaster	*Fruitfly*	KIQNNGD**F**FDLYGGE	F62
	**SHP1**		
H. sapiens		RIQNSGD**F**YDLYGGE	F60

b

Conservation of Y62 in nSH2 domain in *Homo sapiens*

Proteins	Sequence	Phosphosite	Frequency
SHP2	KIQNTGD**Y**YDLYGGE	Y62	2116
RASA1	IIAMCGD**Y**YIGGRRF	Y239	3
YES	RKLDNGG**Y**YITTRAQ	Y222	1816
FYN	RKLDNGG**Y**YITTRAQ	Y213	1819
FGR	RKLDMGG**Y**YITTRVQ	Y208	306
LYN	RSLDNGG**Y**YISPRIT	Y193	787
BLK	RCLDEGG**Y**YISPRIT	Y187	148
SLAP/SLA	FRLPNNW**Y**YISPRLT	Y142	None reported
SH2D1B	IFREKHG**Y**YRIQNSN	Y62	None reported
ITK	TNDNPKR**Y**YVAEKYV	Y305	None reported
BTK	CSTPQSQ**Y**YLAEKHL	Y344	189
TEC	TTTSPKK**Y**YLAEKHA	Y312	None reported

Supplementary Figure 1: Conservation of SHP2 Y62 across animal species. a Sequence alignment of SHP2 orthologs across species, and human SHP1. **b** Sequence alignments of human SH2 domains with phosphosite and frequency indicated.

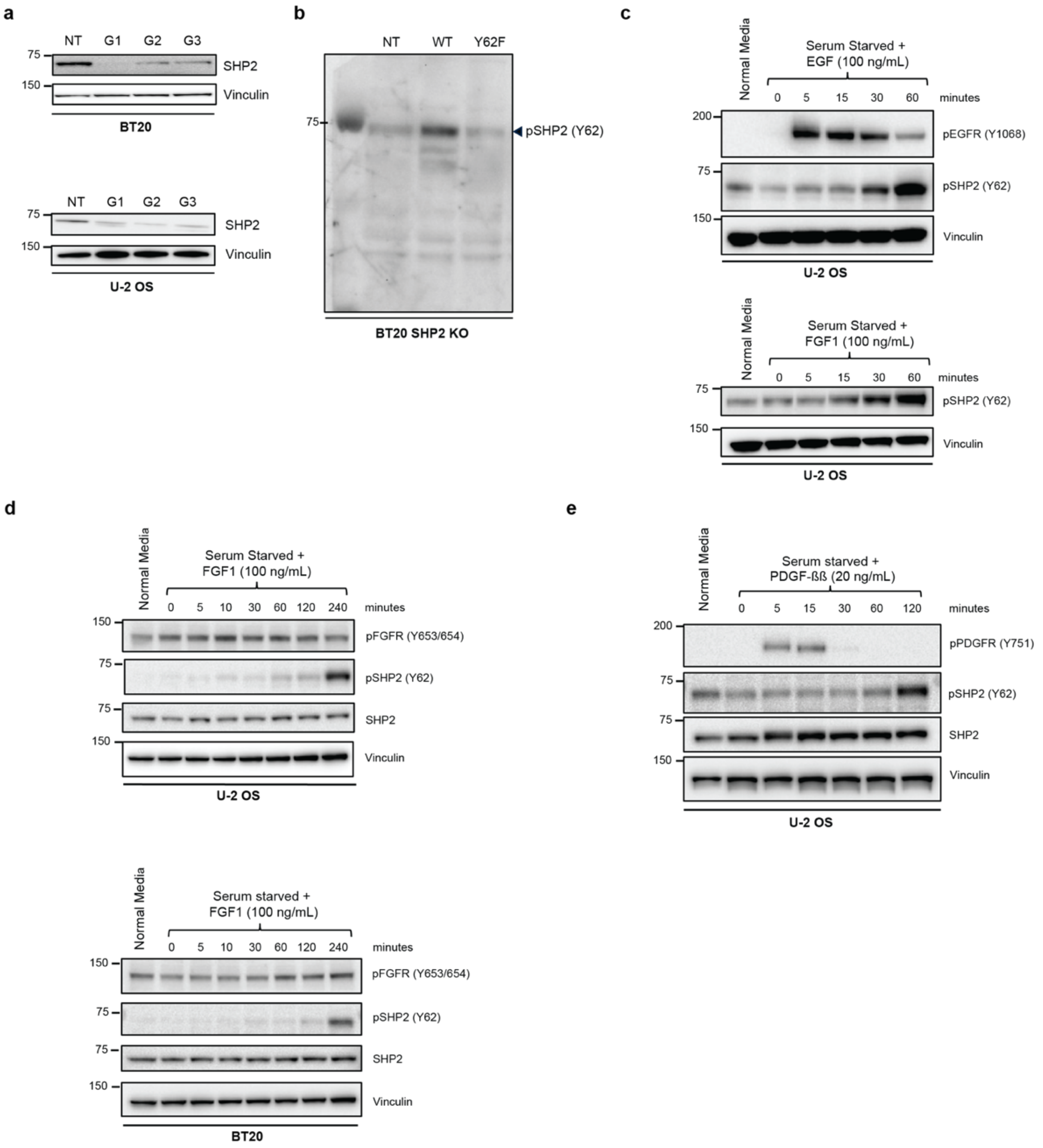

Supplementary Figure 2: SHP2 pY62 is downstream of RTKs. **a** Immunoblot analysis of BT20 and U2-OS cells bearing SHP2 knock out. NT, non-targeting guide RNA. **b** Immunoblot analysis of pSHP2 Y62 antibody in BT20 SHP2 knock out cells overexpressing SHP2^{WT} and SHP2^{Y62F}. **c-e** Immunoblot analysis of indicated cell lines serum starved (24 hours) then treated with (**c**) EGF **(**100 ng/mL), (**d**) FGF1 (100 ng/mL), and (**e**) PDGF-ββ (20 ng/mL) for the indicated timepoints. Source data are provided as source data file. The unit of molecular weight markers in western blots is kDa.

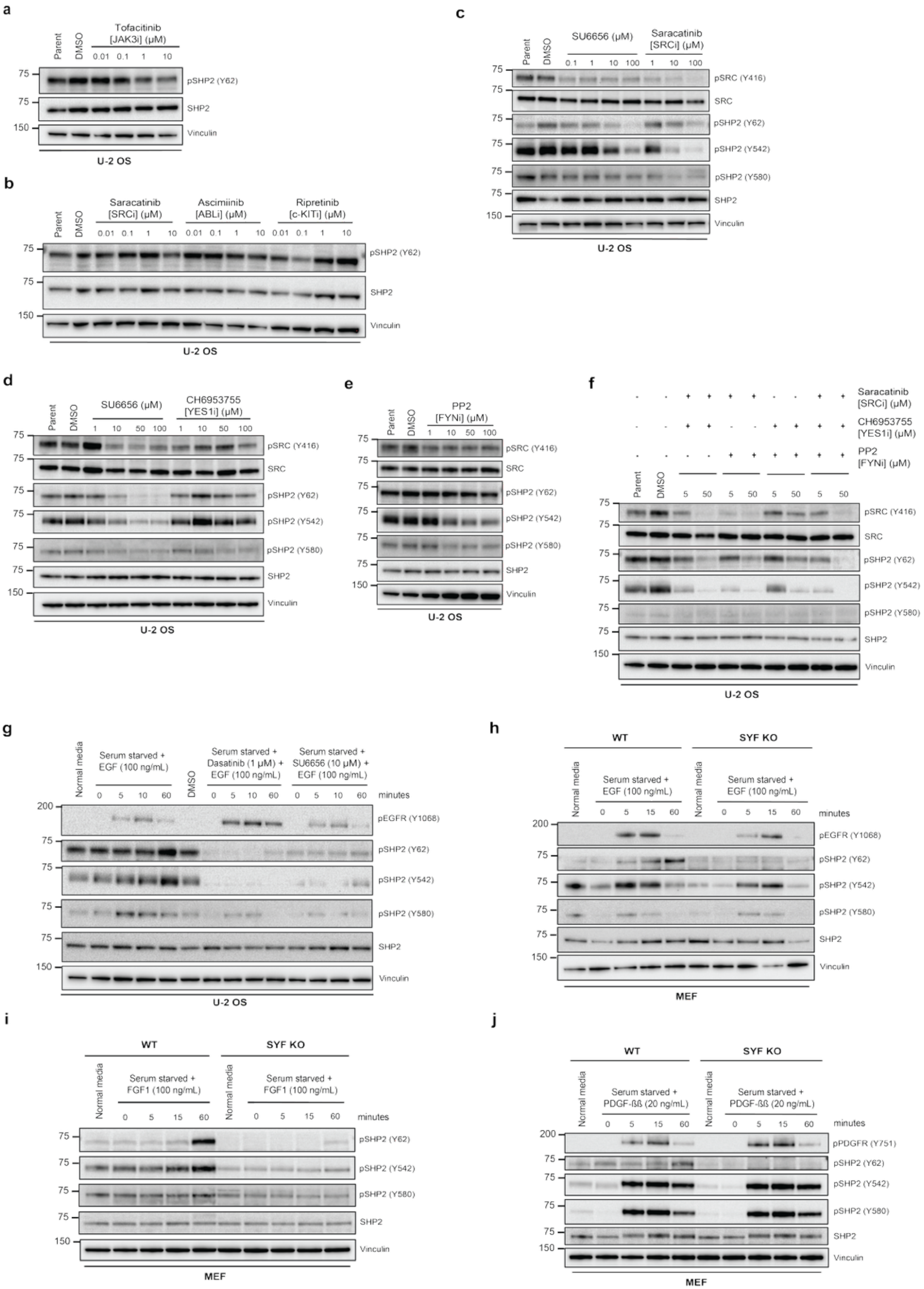
a
Parent
DMSO
Tofacitinib [JAK3i] (µM)
0.01 0.1 1 10
pSHP2 (Y62)
SHP2
Vinculin
U-2 OS
b
Saracatinib [SRCi] (µM)
Ascimiinib [ABLi] (µM)
Ripretinib [c-KITi] (µM)
pSHP2 (Y62)
SHP2
Vinculin
U-2 OS
c
SU6656 (µM)
Saracatinib [SRCi] (µM)
0.1 1 10 100 1 10 100
pSRC (Y416)
SRC
pSHP2 (Y62)
pSHP2 (Y542)
pSHP2 (Y580)
SHP2
Vinculin
U-2 OS
d
SU6656 (µM)
CH6953755 [YES1i] (µM)
1 10 50 100 1 10 50 100
pSRC (Y416)
SRC
pSHP2 (Y62)
pSHP2 (Y542)
pSHP2 (Y580)
SHP2
Vinculin
U-2 OS
e
PP2 [FYNi] (µM)
1 10 50 100
U-2 OS
f
Saracatinib [SRCi] (µM)
CH6953755 [YES1i] (µM)
PP2 [FYNi] (µM)
5 50 5 50 5 50 5 50
U-2 OS
g
Normal media
Serum starved + EGF (100 ng/mL)
Serum starved + Dasatinib (1 µM) + EGF (100 ng/mL)
Serum starved + SU6656 (10 µM) + EGF (100 ng/mL)
0 5 10 60 minutes
pEGFR (Y1068)
U-2 OS
h
WT
SYF KO
Serum starved + EGF (100 ng/mL)
0 5 15 60 minutes
MEF
i
Serum starved + FGF1 (100 ng/mL)
MEF
j
Serum starved + PDGF-ßß (20 ng/mL)
pPDGFR (Y751)
MEF

Supplementary Figure 3: SRC, YES1, and FYN (SYF) kinases phosphorylate SHP2 Y62, Y542, and Y580. a-f Immunoblot analysis of U-2 OS cells treated with (**a**) tofacitinb, (**b**) saracatinib, asciminib, and ripretinib, (**c**) SU6656 and saracatinib, (**d**) SU6656 and CH6953755, (**e**) PP2, (**f**) and double and triple combinations of saracatinib, CH6953755 and PP2 at indicated concentrations, and DMSO. **g** Immunoblot analysis of U-2 OS cells, serum-starved (24 hours) and treated with dasatinib and SU6656, followed by EGF (100 ng/mL) for indicated timepoints. **h-j** Immunoblot analysis of SYF knock out and wildtype MEFs, serum-starved and treated with (**h**) EGF (100 ng/mL), (**i**) FGF1 (100 ng/mL), and (**j**) PDGF-ββ (20 ng/mL). Source data are provided as source data file. The unit of molecular weight markers in western blots is kDa.

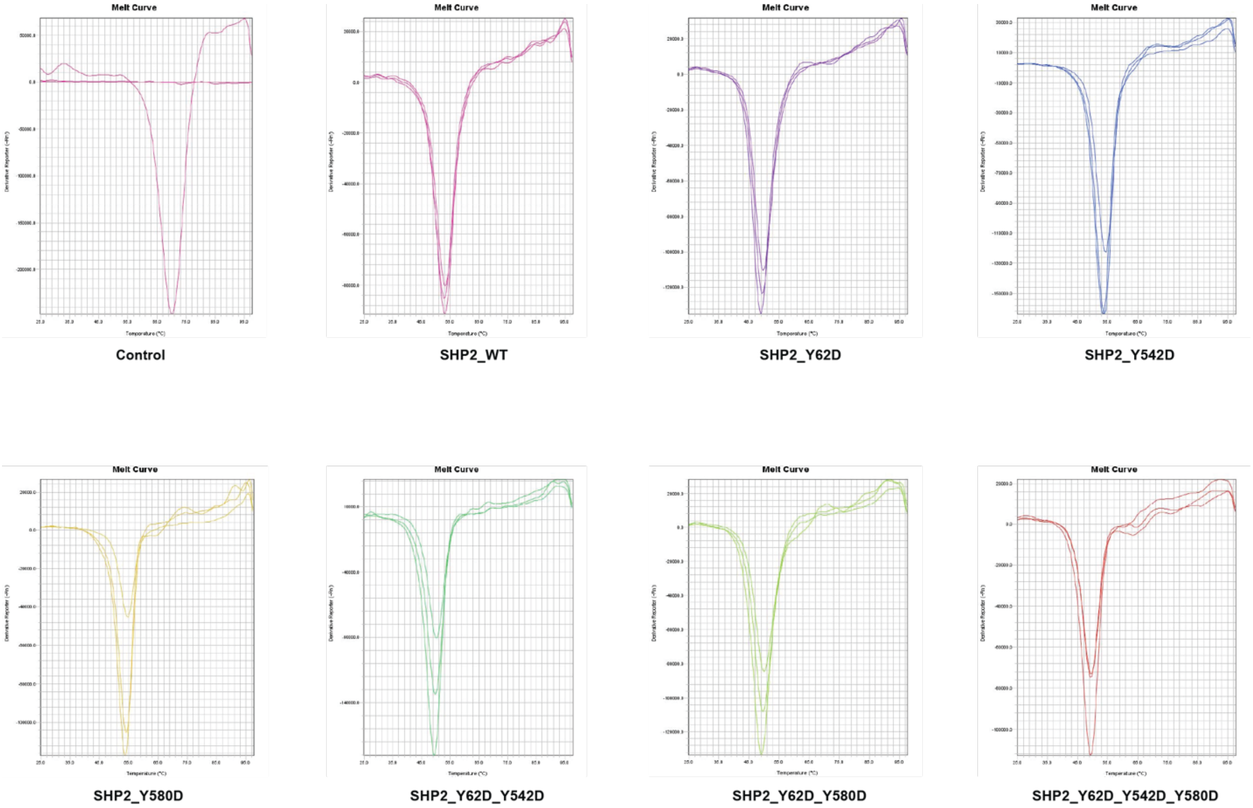

Supplementary Figure 4: Melting temperatures of SHP2 variants. Melting temperatures (T_m) of wild-type and mutant SHP2 proteins by differential scanning fluorimetry (DSF) (n = 3).

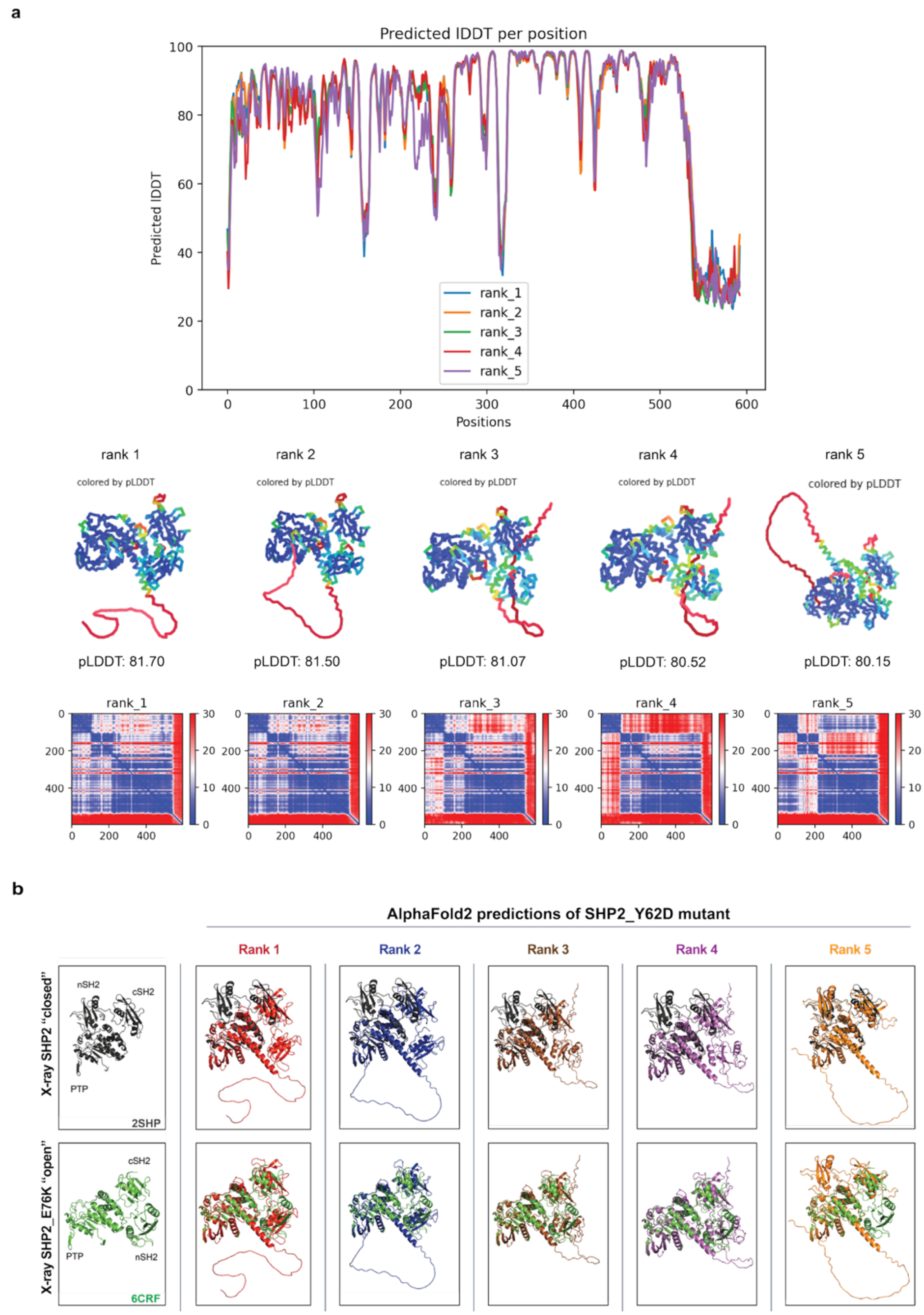

Supplementary Figure 5: AlphaFold2 model predictions of SHP2^{Y62D} mutant structure. **a** Predicted Local Distance Difference Test (pLDDT) scores for the first five ranked predicted SHP2^{Y62D} mutant structure models with their predicted aligned errors (PAE). **b** Superimpositions of first five ranked AlphaFold2-predicted structures of SHP2^{Y62D} mutant onto crystal structures of SHP2^{E76K} (green) (PDB: 6CRF) and SHP2^{WT} (gray) (PDB: 2SHP).

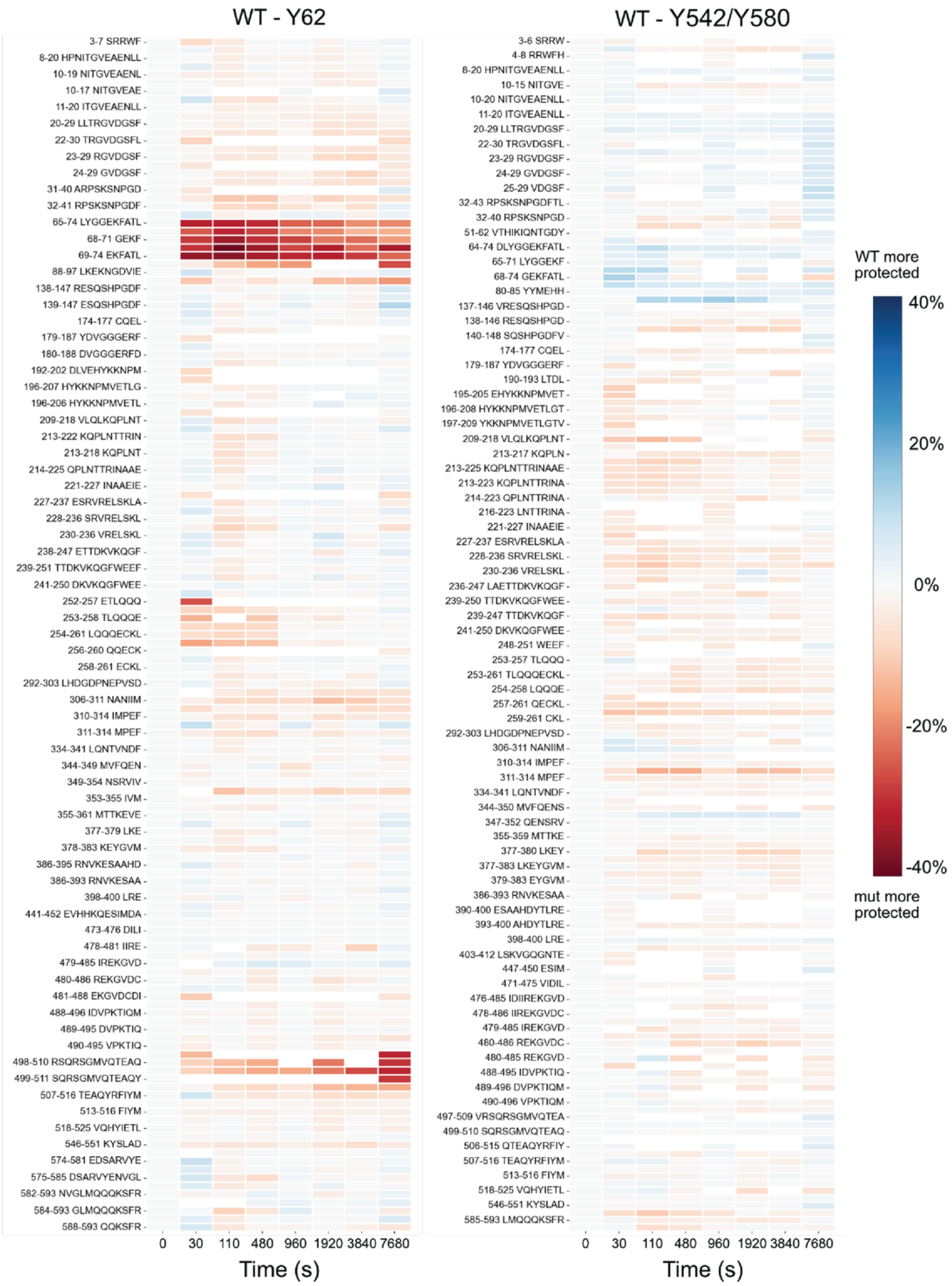

Supplementary Figure 6: Peptide-level exchange vs time-course heat plots of SHP2 mutants Y62D and Y542D/Y580D. Peptide-level exchange vs time-course heat plots comparing SHP2^{WT} to the two SHP2 mutants, Y62D and Y542D/Y580D. Plots show peptide length-normalized exchange at each timepoint. Source data are provided as source data file.

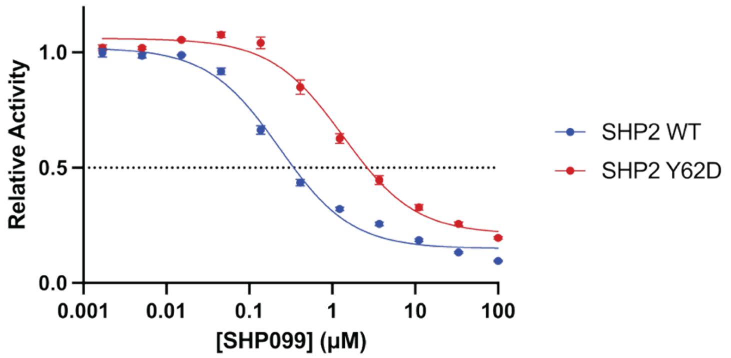

	SHP2 WT	SHP2 Y62D	P value
IC_{50} (μM)	0.2319	1.356	0.0004

Supplementary Figure 7: IC_{50} of recombinant proteins $SHP2^{WT}$ and $SHP2^{Y62D}$ mutant for SHP099. The IC_{50} values of recombinant proteins $SHP2^{WT}$ and $SHP2^{Y62D}$ mutant for SHP099 (0.2319 μM and 1.356 μM, respectively), and the P value (0.0004) measured with two-tailed t test (n=4, 95 % confidence interval, effect size=0.8933, degree of freedom=6). Data are presented as mean values +/- SEM. Source data are provided as source data file.

Supplementary Protocol – Gibson cloning of SHP2 mutants into pLX304

Overview

The protocol consists of 3 PCR steps and 1 Gibson reaction

<u>PCR 1: Amplifies part 1 of the pLX304 backbone (RRID:Addgene_25890)</u>

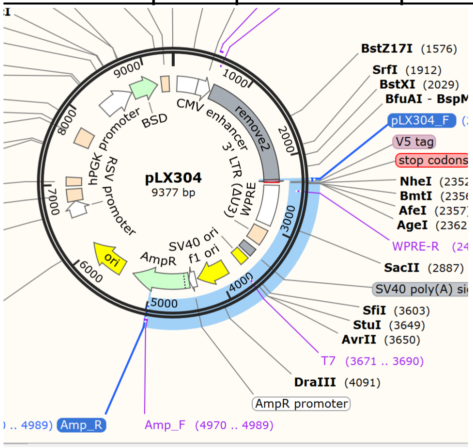

<u>PCR 2: Amplifies part 2 of the pLX304 backbone</u>

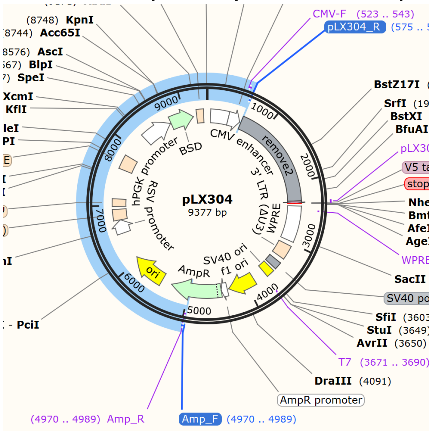

<u>PCR 3: Amplifies the SHP2 variants of the pGEX vectors (Addgene, Cat# 27-4584-01)</u>

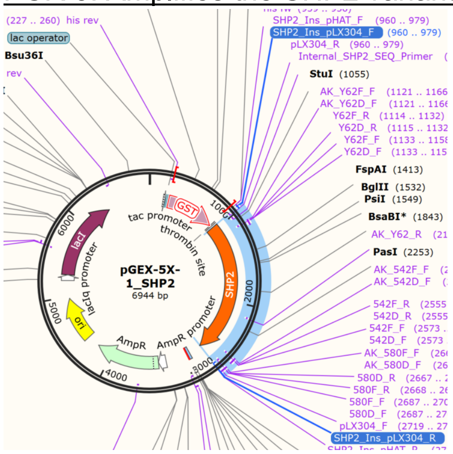

Materials
Gibson 2x MM
Primers
- Amp_F
- Amp_R
- pLX304_F
- pLX304_R
- SHP2_Ins_pLX304_F
- SHP2_Ins_pLX304_R

Plasmids
- pLX304
- pGEX_SHP2^{WT}
- pGEX_SHP2^{Y62F}
- pGEX_SHP2^{Y542F}
- pGEX_SHP2^{Y580F}
- pGEX_SHP2^{Y62F_Y542F}
- pGEX_SHP2^{Y62F_Y580F}
- pGEX_SHP2^{Y542F_Y580F}
- pGEX_SHP2^{Y62F_Y542F_Y580F}

1% Agarose gel
DpnI (NEB, Cat# R0176S)
PCR clean up kit (Macherey-Nagel, Cat# 740609)
Gibson 2x HIFI MM (NEB, Cat# E2611L)
E. coli Dh5alpha (NEB, Cat# C2987H)

Methods
PCR 1 – 200 µL – **2690 bp** – 68 degrees annealing

Prepare in a PCR tube:

97 uL Nuclease Free Water
1 uL pLX304**_F** (100 µM)
1 uL Amp**_R** (100 µM)
1 uL pLX304 S (~200 ng/µL)
100 uL Q5 2x MM

Vortex – spin down – Divide 100 µL over 2 PCR tubes

PCR: 30 cycles of step 2-4
98 30 seconds
98 10 seconds
68 15 seconds
72 **1.5** minutes
72 2 minutes
10 hold

PCR 2 – 200 uL – **5000 bp** – 68 degrees annealing

Prepare in a PCR tube:

97 µL Nuclease Free Water
1 µL pLX304**_R** (100 µM)
1 µL Amp**_F** (100 µM)
1 µL pLX304 S (~200 ng/µL)
100 µL Q5 2x MM

*Really watch that you are adding the 1 µL!

Vortex – spin down – Devide 100 µL over 2 PCR tubes

PCR: 30 cycles of step 2-4
98 30 seconds
98 10 seconds
68 15 seconds
72 **5** minutes
72 **10** minutes
10 hold
PCR 3 – 8x 100 µL – **1783 bp** – 68 degrees annealing
Prepare MM in a 1.5 mL tube:

388 µL Nuclease Free Water

4 µL SHP2_Ins_pLX304_**F** (100 µM)
4 µL SHP2_Ins_pLX304_**R** (100 µM)
400 µL Q5 2x MM

Vortex – spin down - Devide over 8 PCR tubes: 98 µL each (just to make sure you don't have to little for the last one)

Add 0.5 µL of each pGEX SHP2 plasmid to a tube – watch closely.

pGEX_SHP2^{WT} = 1
pGEX_SHP2^{Y62F} = 2
pGEX_SHP2^{Y542F} = 3
pGEX_SHP2^{Y580F} = 4
pGEX_SHP2^{Y62F_Y542F} = 5
pGEX_SHP2^{Y62F_Y580F} = 6
pGEX_SHP2^{Y542F_Y580F} = 7
pGEX_SHP2^{Y62F_Y542F_Y580F} = 8

Vortex – spin down

PCR: 30 cycles of step 2-4
98 30 seconds
98 10 seconds
67 15 seconds
72 **1** minutes
72 2 minutes
10 hold

<u>1% Agarose gel control</u>
Take 5 µL of each unique sample (so for PCR 1 and PCR2 only 1 check, for PCR 3 all 8 samples) to a gel – check if the size is roughly correct.

5 µL PCR
5 µL H2O
2 µL 6x Loading dye

Left over 95 µL PCR can be stored at 4 degrees if not immediately continuing to DpnI treatment.

<u>DpnI digestion</u>

To each PCR tube, add 1 µL DpnI
Incubate at 37 degrees in the PCR machine for 30 minutes

<u>PCR clean up</u>

Immediately following DpnI digest – clean up the PCR/DpnI samples

Use Nucleaspin Gel and PCR clean up

For PCR 1/2
Add 400 µL yellow binding buffer to a 1.5 mL tubes
Combine and add 200 µL PCR reaction

For PCR 3
Add 200 µL yellow binding buffer to a 1.5 mL tubes (1 for each PCR tube)
Add 100 µL PCR reaction

Follow protocol. Elute with nuclease free water. Elute 20 µL for PCR 1 and 2, 15 µL for each PCR 3.

<u>Measure concentrations – can be stored at -20.</u>

<u>Gibson</u>

*Volumes depend on concentrations of DNA – lets decide when you know.

In PCR tube

1 µL Backbone PCR1
2 µL Backbone PCR2
1 µL SHP2 Insert DNA
5 µL 2x HIFI MM

Incubate for 1 hour at 50 degrees Celsius (lid at 80 degrees).

Gibson can be stored at -20 if not immediately continuing to transformation.

<u>Transformation</u>
Transform 2.5 µL to 50 µL dh5x cells

As per protocol (30 seconds at 42 heatshock)

Plate all cells to ampicillin plates.
