# Supplementary material for: A hotspot phosphorylation site on SHP2 drives oncoprotein activation and drug resistance
Source: Nat Commun. 2026 Mar 3;17:3383. doi: 10.1038/s41467-026-70060-8 (PMC13066003; doi:10.1038/s41467-026-70060-8)
Supplement: Supplementary file 3 — Description of Additional Supplementary Files [file 41467_2026_70060_MOESM3_ESM.pdf]

Description of Additional Supplementary Files

Supplementary Data 1: Phosphosite frequency.

Supplementary Data 2: NSCLC protein enrichment.

Supplementary Data 3: Kinase library predictions

Supplementary Data 4: Predicted AlphaFold2 models.
