# Supplementary material for: A hotspot phosphorylation site on SHP2 drives oncoprotein activation and drug resistance
Source: Nat Commun. 2026 Mar 3;17:3383. doi: 10.1038/s41467-026-70060-8 (PMC13066003; doi:10.1038/s41467-026-70060-8)
Supplement: Supplementary file 4 — Transparent Peer Review file [file 41467_2026_70060_MOESM4_ESM.pdf]

nature portfolio

Peer Review File

A Hotspot Phosphorylation Site on SHP2 Drives Oncoprotein Activation and Drug Resistance

Corresponding Author: Dr Neil Vasan

This file contains all reviewer reports in order by version, followed by all author rebuttals in order by version.

Version 0:

Reviewer comments:

Reviewer #1

(Remarks to the Author)
Karunaraj, P., Scheele, R., et al. characterized biochemical and structural properties of the SHP2 Y62 phosphorylation using the Y62D phosphomimetic mutant. The authors' bioinformatics study attempted to substantiate the importance of the Y62 phosphorylation. Using various kinase inhibitors, the authors identified SRC and other SFKs as the kinases that directly phosphorylate SHP2 at Y62.
I appreciate that the authors employed multidisciplinary approaches to convey their point. However, there is insufficient evidence for SHP2 Y62 phosphorylation by SRC and other SFKs, as the authors claim. This is because the inhibitor studies presented (Fig. 2, 3, and Supplemental Fig. 3) lack essential data; none of these blots display the amount of the inhibitor-targeted kinases. For example, the authors should include a blot showing depletion of active pY1068 EGFR with erlotinib treatment, alongside pY62 SHP2 (Fig. 2c). All the authors' inhibitor studies suffer from the same problem. The authors must show that the added inhibitors had the intended effect on targeted kinases (decrease/disappearance of the active RTKs/SFKs) along with the level of SHP2 phosphorylation.
Also, where is the experimental evidence for pY62 being a drug-resistant mutation? Was there a previous study determining IC50 for the pY62 mutant against SHP2 inhibitors? If not, the authors should determine IC50 against one representative SHP2 inhibitor to make the title of this manuscript valid.
The authors presented Fig.6 as evidence for pY62 drug resistance. This suffers from the same issue as Figs. 2 and 3 as stated above. Fig.6B-D does not show phospho-EGFR level with EGF and the level of SHP2 after the transfection. What happened to the level of SHP2 with each inhibitor?
Other points are the following:
Figure 1B-1G: The content of these figures is not clear at all.
Fig.1B and 1C: What does the Y-axis of the graph correspond to? I understand that the authors identified a total of 239,531 phosphosites in all human proteins combined, but why are there multiple hits at y-axis (hits) = 1? Do these correspond to proteins with 1 phospho-sites? But, that doesn't make sense since I see a lot of points above 100 hits. In summary, what does the "hit" correspond to? The authors must describe this point clearly in the manuscript.
Fig.1C and 1D:
Line 107-110: These statements appear to be contradictory. According to the authors, SHP2 pY62 is the most abundant in the database search, but pY62 is less reported than the canonical pY542 and pY580. Please provide a proper explanation on this point.
Fig.1E-1G: The authors found pY62 SHP2 as the most abundant phosphorylation in different cancer cell lines except for KRAS-mutant NSCLC. Considering tyrosine phosphorylation comprises only ~ < 1-2 % of the entire proteome at most, the authors' finding is pretty shocking. Also, the phosphorylation abundance of different studies cannot be compared since different sample preparation methods, LC, as well as mass spectrometry settings, are variable and influence the determined abundance (or amount) of phosphopeptides.
If the authors wish to highlight the significance of pY62, a previous study (ref 30) seems to be the most suitable for elaboration, instead of the Figure 1 results.
As to Reference 30, this study found that FLT3 phosphorylates SHP2 at Y62, as opposed to the authors' finding. Please elaborate on this point.
Figures 2 and 3: As stated on the first page, the level of inhibitor-targeted kinases should be shown on blots. Also, Fig.3a is difficult to see.
Figure 4B: This is probably the most interesting result in this manuscript. The Y62D mutant becomes insensitive to the presence of an allosteric peptide activator, while the peptide can activate the other mutants. Where is the peptide binding

site?
Figure 5A: Structural alignment between an AlphaFold predicted model and a crystal structure of SHIP2 in a closed conformation (top right).
• The figure does not appear to make sense. The PTP domain appears very similar in both structures, with the only difference being the orientation of the SH2 domain. Did AlphaFold provide alternative structures that closely resemble the closed conformation?
Figure 5C: The percent exchange structure map
The preparation of the fully-deuterated standard poses an issue. The point of doing a fully-deuterated experiment is to obtain the maximum deuterium uptake of each peptide under the same labeling strength (pH, temperature, and deuterium dilution factor) as that of the labeling experiment. This allows us to normalize the deuterium uptake of each peptide and express them as the percent exchange. This is important since the structural flexibility of different regions in the same state can be compared. For example, the structural flexibility of region A and region B in the same protein (state) can be compared since a fully-deuterated experiment corrects for the back-exchange difference between the two regions (corrects for back-exchange dependence on amino acid composition/sequence of peptides). It also enables the comparison of the percent exchange between different proteins, provided that the fully-deuterated experiments are appropriately performed.
The labeling strength is highly dependent on labeling temperature. So, the authors' result vastly underestimates structural flexibility due to the higher temperature employed for the full-deuterated experiment. For example, consider the following scenario where you are analyzing a 10-residue peptide. The maximum possible uptake is 9 (no proline), and you want to determine the fully-denatured/deuterated uptake of this peptide. The labeling strength is lower at 15 °C than at 37 °C. So, the experimentally determined maximum uptake of all peptides will be higher at 37 °C (Say Duptake = 7 for this peptide) than at 15 °C (Duptake = 5). If you then determine the same peptide uptake to be 3, the calculated percent exchange at 37 °C and 15 °C is 43% and 60%, respectively. A viable remedy for this issue is to correct the deuterium uptake data using the different temperature factor (or repeat the fully-deuterated experiments).
Alternatively, since Fig.5C uptake plots (in Da, the same peptide uptake comparisons) are appropriate, I suggest that the authors keep these exchange uptake data. It will be helpful if the authors indicate/highlight peptide regions in the SHP2 structure more clearly.
Also, based on the HDX-MS results, can the authors speculate how the pY62 mutant (Y62D) confers drug resistance? Is the Y62D mutant drug resistant like the pY62 mutant?
In summary, the title of this manuscript does not align with the content. The manuscript requires major revision, with a substantial number of additional experiments needed to show a connection between pY62 activation and drug resistance.

Reviewer #2

(Remarks to the Author)
This paper reports a thorough exploration of phosphorylation of SHP2 on Y62 as a molecular mechanism of sustaining SHP2 in an active form and as a mechanism of resistance to SHP2 inhibition. The data generally support the conclusions drawn by the authors. Overall, the work will be a meaningful contribution to the field, and I am supportive of publication after the refinement of the x-ray structure is completed (see below) and the authors make the requisite textual edits to give the prior work proper recognition.

Major points:

1) The authors unnecessarily downplay the prior identification of pY62 that was reported in two other manuscripts. They do cite both papers, but their text in the introduction and discussion minimizes the value of the earlier work and makes it appear as if the authors are making a breakthrough discovery of Y62 phosphorylation. They should be more forthright in acknowledging the prior work documenting Y62 phosphorylation as a resistance mechanism. What makes this work stand out is that it more fully explores the generality of Y62 phosphorylation across cancer cell types and the scope of contexts in which the modification may impact signaling, reports a pY62 phosphoselective Ab to probe direct versus indirect induction of the pY mark, thereby assigning phosphorylation to SFKs, rather than the RTKs, and uses Y62D as a phosphomimetic for structural analysis, HX-MS, and biochemical studies to complement the cellular work.

2) Review of the x-ray structure table, text, pdb validation report, model, and map shows that the claim in the table of 2.63 Å resolution is not congruent with the text, which states a resolution of 2.9 Å, and that the data set is not complete, particularly in the outermost shell. The Rwork and Rfree values are unexpectedly high, even for the claimed 2.9 Å resolution, and the quality of the map and model fit could be substantially improved. Attention to the refinement might also clarify whether there could be a bridging H_2O molecule between D61 and D62.

One other minor point - the enzymatic data to measure catalytic parameters appear to substantially overestimate the activity of the WT (unmutated) protein compared to values published elsewhere. The basal activity of wt SHP2 is difficult to measure accurately, but it is not clear why that would be true.

Reviewer #3

(Remarks to the Author)
SHP2 is a phosphatase and a critical mediator of receptor tyrosine kinase (RTK)-driven RAS/mitogen-activated protein kinase (MAPK) signaling. Despite promising preclinical data, SHP2 inhibitors have shown minimal clinical efficacy, with no defined clinical mechanisms of primary resistance. Therefore, it is important to elucidate the underlying mechanism on the lack of clinical response of SHP2 inhibitors in human cancer and to develop new therapeutic strategies to target SHP2.

In this study, the authors elucidated phosphorylation of SHP2 at tyrosine 62 (pY62) as a hotspot phosphorylation site in the proteome and RTK-driven tumor types in patients. They demonstrated that SRC family kinases directly phosphorylate SHP2 at Y62, downstream of but not directly phosphorylated by RTKs. Using biochemical and biophysical analyses, the authors showed that SHP2 Y62D (a phosphor tyrosine mimic) enforces an open, active conformation, resulting in constitutive phosphatase activation that is sufficient to activate MAPK signaling and confer resistance to allosteric SHP2 inhibitors. Their findings establish that SHP2 pY62 is a phosphorylation hotspot phenocopying mutational activation, a mechanism of primary resistance to SHP2 inhibitors, and a cancer drug target distinct from wildtype SHP2.

This study employed a number of complementary approaches and the experiments were well done. The conclusion was solid and well justified. The manuscript was well written and easy to follow. This study answered an urgent and important question for the SHP2 filed and provided novel therapeutic approaches to target active SHP2, instead of inactive SHP2. It is recommended that the study be published in Nature Communications.

Version 1:

Reviewer comments:

Reviewer #1

(Remarks to the Author)
I thank the authors for taking considerable time to address my concerns. I would also like to commend them for performing through experiments and for providing a clear and thoughtful rebuttal to my comments.

I would like to offer one additional point regarding HDX-MS analysis. Comparison of percent deuterium exchange across different peptides is appropriate provided that fully deuterated control experiments are performed. In response, the authors state that
"back-exchange correction alone does not make peptides with different amino-acid compositions directly comparable,because each sequence has its own intrinsic exchange rates that cannot be precisely normalized on a residue-by-residue basis."

However, the purpose of back-exchange correction is precisely to normalize for sequence-dependent effect, including intrinsic exchange arising from amino acid composition. Indeed, examination of back-exchange values across peptides often reveal clear sequenced dependence – for example, histidine-containing peptides typically show higher back exchange – demonstrating that such correction explicitly account for intrinsic sequence effects. Once normalized using fully deuterated controls, percent deuterium exchange therefore allows meaningful comparison between different peptides.

If percent exchange across peptides were fundamentally invalid, the utility of back-exchange correction would be unclear. In that case, analysis would be restricted to absolute deuterium uptake (Da), limiting comparisons to identical peptides across conditions and substantially reducing the interpretive power of HDX-MS.

Furthermore, full deuteration can be reliably achieved for most soluble proteins within 1-3 minutes at 25 C using high denaturant concentration (e.g., 6 M guanidine-DCl, or 8 M urea-d4), provided that the labeling strength is sufficient (~20-fold deuterium dilution). I recommend the authors to visit: https://hx2.med.upenn.edu/download.html, which calculates theoretical intrinsic exchange as well as exchange rates in various conditions (pH, temp). Although the site only calculates these rates for given peptide sequences, it may be useful to estimate full exchange times.

Reviewer #2

(Remarks to the Author)
The authors adequately addressed my comments about the referencing of prior work, their X-ray data, and the catalytic activity of the autoinhibited WT enzyme. I defer to the first reviewer regarding comments about SFKs as the kinases responsible for Y62 phosphoryation, as well as their other concerns.

Open Access This Peer Review File is licensed under a Creative Commons Attribution 4.0 International License, which permits use, sharing, adaptation, distribution and reproduction in any medium or format, as long as you give appropriate credit to the original author(s) and the source, provide a link to the Creative Commons license, and indicate if changes were made.
In cases where reviewers are anonymous, credit should be given to 'Anonymous Referee' and the source.
The images or other third party material in this Peer Review File are included in the article's Creative Commons license, unless indicated otherwise in a credit line to the material. If material is not included in the article's Creative Commons license and your intended use is not permitted by statutory regulation or exceeds the permitted use, you will need to obtain permission directly from the copyright holder.
To view a copy of this license, visit https://creativecommons.org/licenses/by/4.0/

Reviewer #1 (Remarks to the Author):

Karunaraj, P., Scheele, R., et al. characterized biochemical and structural properties of the SHP2 Y62 phosphorylation using the Y62D phosphomimetic mutant. The authors' bioinformatics study attempted to substantiate the importance of the Y62 phosphorylation. Using various kinase inhibitors, the authors identified SRC and other SFKs as the kinases that directly phosphorylate SHP2 at Y62. I appreciate that the authors employed multidisciplinary approaches to convey their point.

We thank the reviewer for acknowledging our multidisciplinary approach.

However, there is insufficient evidence for SHP2 Y62 phosphorylation by SRC and other SFKs, as the authors claim. This is because the inhibitor studies presented (Fig. 2, 3, and Supplemental Fig. 3) lack essential data; none of these blots display the amount of the inhibitor-targeted kinases. For example, the authors should include a blot showing depletion of active pY1068 EGFR with erlotinib treatment, alongside pY62 SHP2 (Fig. 2c). All the authors' inhibitor studies suffer from the same problem. The authors must show that the added inhibitors had the intended effect on targeted kinases (decrease/disappearance of the active RTKs/SFKs) along with the level of SHP2 phosphorylation.

We thank the reviewer for this comment. To address the need for on-target validation, we performed immunoblots for active RTKs and SFKs under the same treatment conditions used in all inhibitor experiments. pEGFR, pFGFR, pPDGFR, and pSRC (Y416) blots now accompany each assay in the revised Figures 2 and 3 and Supplemental Figure 3, confirming effective suppression of the targeted kinases. We note that the polyclonal pSRC (Y416) antibody (CST #2101) cross-reacts with pYES and pFYN.

As representative examples, erlotinib treatment markedly reduces pEGFR (Y1068) in the same samples used for pY62 SHP2 analysis (revised Fig. 2C), and dasatinib reduces pSRC (Y416) under conditions used for SFK inhibition (revised Fig. 3C).

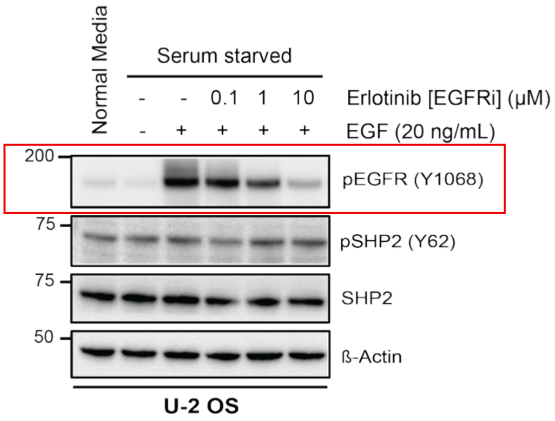

Revised Figure 2c

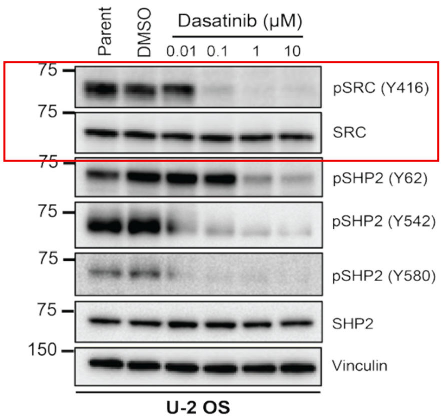

Revised Figure 3c

- Also, where is the experimental evidence for pY62 being a drug-resistant mutation? Was there a previous study determining IC50 for the pY62 mutant against SHP2 inhibitors? If not, the authors should determine IC50 against one representative SHP2 inhibitor to make the title of this manuscript valid.

We thank the reviewer for this question. We are not aware of any prior studies reporting SHP2 inhibitor IC_{50} values for pY62 or its phosphomimetic Y62D. Therefore, we performed biochemical inhibition assays using purified recombinant SHP2 WT and Y62D proteins. SHP099 IC_{50} values were 0.2319 μM for WT and 1.356 μM for Y62D (Supplementary Fig. 7), a statistically significant shift. These data show that the Y62D variant has reduced sensitivity to allosteric SHP2 inhibition.

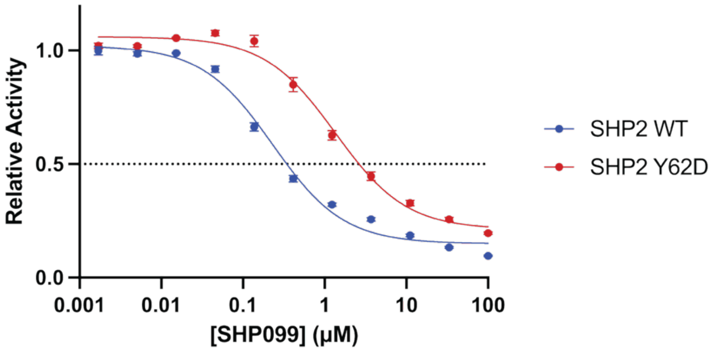

	SHP2 WT	SHP2 Y62D	P value
IC_{50} (μM)	0.2319	1.356	0.0004

Supplementary Figure 7

- The authors presented Fig.6 as evidence for pY62 drug resistance. This suffers from the same issue as Figs. 2 and 3 as stated above. Fig.6B-D does not show phospho-EGFR level with EGF and the level of SHP2 after the transfection. What happened to the level of SHP2 with each inhibitor?

We thank the reviewer for this comment. We have added blots for pEGFR (Y1068) and total SHP2 to the revised Figure 6B-D. As shown in the revised panels, pEGFR increases appropriately with EGF stimulation, and total SHP2 levels remain unchanged across the isogenic lines, indicating that the observed differences are not attributable to altered SHP2 expression. We have shown Revised Fig. 6B as a representative example here:

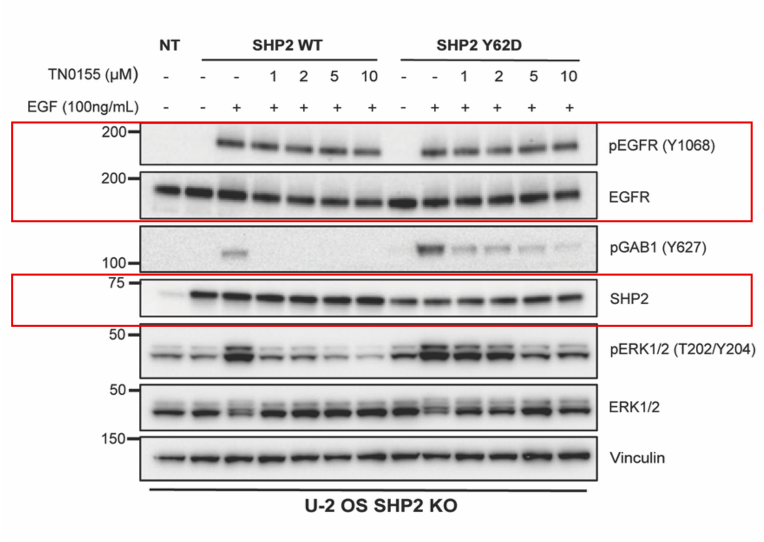

Revised Figure 6b

Other points are the following:
Figure 1B-1G: The content of these figures is not clear at all.

- Fig.1B and 1C: What does the Y-axis of the graph correspond to? I understand that the authors identified a total of 239,531 phosphosites in all human proteins combined, but why are there multiple hits at y-axis (hits) = 1? Do these correspond to proteins with 1 phospho-sites? But, that doesn't make sense since I see a lot of points above 100 hits. In summary, what does the "hit" correspond to? The authors must describe this point clearly in the manuscript.

We thank the reviewer for pointing out this lack of clarity. In Figures 1B and 1C, the Y-axis ("hits") represents the sum of two PhosphoSitePlus annotations: MS_LIT, the number of published studies reporting a given phosphosite, and MS_CST, the number of unpublished mass-spectrometry experiments curated by Cell Signaling Technology. Most sites have only a single hit, typically from MS_CST, whereas higher values reflect sites detected repeatedly across many studies or datasets.

We have removed the y-axis label "hits" and have relabeled the y-axis to "MS_LIT + MS_CST" in Figure 1B and 1C. The definitions covering MS_LIT and MS_CST have now been added to the Results and Methods sections to ensure this point is clear in the manuscript.

- Fig.1C and 1D:
  Line 107-110: These statements appear to be contradictory. According to the authors, SHP2 pY62 is the most abundant in the database search, but pY62 is less reported than the canonical pY542 and pY580. Please provide a proper explanation on this point.

We thank the reviewer for highlighting this point. In Figure 1D, we quantified the characterization frequency in the literature, using (LT_LIT + 1) / (MS_LIT + MS_CST). LT_LIT, another metric reported on PhosphoSitePlus, counts only "low-throughput" (LT), experimentally validated

studies and represents a small subset of MS_LIT. In the numerator, we added 1 so that phosphosites without LT_LIT entries (which are the majority of phosphorylation sites in PhosphoSitePlus) would still appear on the plot rather than being assigned a value of zero.

Under this metric, SHP2 pY62 has limited experimental characterization (LT_LIT = 1) despite frequent detection in large data sets, whereas pY542 and pY580 have far more LT-validated reports (LT_LIT = 46 and 20, respectively). Above, we discussed how MS_LIT + MS_CST, the denominator here, is higher for pY62 and lower for pY542 and pY580. Together, this explains the lower value for (LT_LIT + 1) / (MS_LIT + MS_CST) for pY62 in Figure 1D and reflects its limited targeted experimental follow-up, rather than a contradiction with Figure 1C. The definition covering LT_LIT has now been added to the Results and Methods sections to ensure this point is clear in the manuscript.

- Fig.1E-1G: The authors found pY62 SHP2 as the most abundant phosphorylation in different cancer cell lines except for KRAS-mutant NSCLC. Considering tyrosine phosphorylation comprises only ~ < 1-2 % of the entire proteome at most, the authors' finding is pretty shocking. Also, the phosphorylation abundance of different studies cannot be compared since different sample preparation methods, LC, as well as mass spectrometry settings, are variable and influence the determined abundance (or amount) of phosphopeptides. If the authors wish to highlight the significance of pY62, a previous study (ref 30) seems to be the most suitable for elaboration, instead of the Figure 1 results.

We thank the reviewer for this thoughtful comment. We agree that global phosphotyrosine abundance is low (<1-2% of all phosphorylation) and that absolute comparisons of phosphosite abundance across studies are not possible due to differences in sample preparation, enrichment, chromatography, and MS settings. In Figures 1E-G, our analysis relies only on within-dataset relative abundance (mutant versus WT tumors), using CPTAC-provided phosphosite intensity values that are not normalized to total protein levels. Our conclusions therefore reflect relative differences within each dataset rather than absolute phosphosite stoichiometry.

We also agree that enrichment methods influence detectability. CPTAC uses pY-specific antibody enrichment for tyrosine sites and IMAC/TiO_2 for serine/threonine sites, so pY and pS/pT values are not directly comparable. However, this methodological distinction primarily affects which peptides enter the dataset, not the rank-ordering of relative abundance *within* a given enrichment workflow. Importantly, because our analysis relies only on within-dataset relative abundances, these methodological differences do not affect the conclusions drawn. Notably, in KRAS-mutant NSCLC—the one context where pY62 is not top-ranking—the most abundant phosphopeptide is a serine/threonine site (KRT8 pS475/pS478), indicating that the observed pattern is not simply an artifact of pY enrichment.

Finally, we appreciate the suggestion to reference the earlier mechanistic study (ref. 30), which we have described more extensively in our Introduction and Discussion. That work identified pY62 in the context of SHP099 resistant FLT3-driven AML cells. Our analysis is complementary:

it arises from an unbiased, multi-dataset, multi-cancer interrogation of cell and tumor phosphoproteomic data, revealing pY62 as a recurrent and relatively enriched pY site across diverse tumor contexts. Thus, Figure 1 provides independent large-scale support for the biological relevance of pY62, which aligns with but does not duplicate the findings of ref. 30.

- As to Reference 30, this study found that FLT3 phosphorylates SHP2 at Y62, as opposed to the authors' finding. Please elaborate on this point.

We thank the reviewer for raising this point. We respectfully note that the data presented in Reference 30 do not demonstrate direct phosphorylation of SHP2 Y62 by FLT3, as suggested in its Abstract. In that study, FLT3-ITD autophosphorylation and SHP2 pY62 both increase in FLT3-ITD AML models of acquired SHP099 resistance, but the experiments do not test whether FLT3 directly phosphorylates Y62. Specifically, SHP2 pY62 levels were not assessed following FLT3 inhibition, and no in vitro kinase assays were performed to demonstrate FLT3-dependent phosphorylation of SHP2.

What Reference 30 does show is a correlation between FLT3 activation and elevated SHP2 pY62. This observation is fully consistent with our model: we likewise find that SHP2 pY62 increases with RTK activation, but our biochemical, kinase, and inhibitor data indicate that the phosphorylation event is mediated by SFKs rather than by the RTKs themselves.

- Figures 2 and 3: As stated on the first page, the level of inhibitor-targeted kinases should be shown on blots. Also, Fig.3a is difficult to see.

We have now included the levels of all inhibitor-targeted kinases as measured by decreased autophosphorylation in the revised blots for Figures 2 and 3, as requested in the first page of the review. In addition, Figure 3A has been enlarged and reformatted to improve clarity and to provide a more easily interpretable visualization of the predicted kinases within the kinome tree.

- Figure 4B: This is probably the most interesting result in this manuscript. The Y62D mutant becomes insensitive to the presence of an allosteric peptide activator, while the peptide can activate the other mutants. Where is the peptide binding site?

We thank the reviewer for this insightful comment. SHP2-activating phosphopeptides bind the canonical pY-binding cleft of the nSH2 (high-affinity) and cSH2 (low-affinity) domains (Eck, *Nature* 1996). We are not aware of any crystal structures of human SHP2 nSH2 bound to pIRS1 (Y1172), which was the peptide used in our experiments (characterized in Fujimoto, *JBC* 1994). Structures of Syp (mouse SHP2) nSH2 bound to pIRS1 (Y895) and pPDGFR (Y1009) as well as structures of human SHP2 nSH2 bound to pPDGFR (Y1009) have demonstrated that the phosphopeptides engage SHP2 nSH2 residues including R32, S33-N36, T42, T52-I56, L65-G68, Y81, L88-K91, and I96 (Lee, *Structure* 1994; Hof, *Cell* 1998; Eck, *Nature* 1996). These interactions stabilize the open, active conformation by relieving nSH2-mediated autoinhibition.

Y62, however, is not part of the peptide-binding surface. It lies instead at the nSH2-PTP interface, where phosphorylation—or the Y62D phosphomimetic—destabilizes autoinhibition and constitutively favors the open state. As a result, Y62D is already “activated” and cannot be further stimulated by the pIRS1 peptide, whereas other mutants retain an autoinhibited ground state that the peptide can still unlock.

Thus, the peptide binds the established nSH2 interface, while Y62D activates SHP2 through a distinct structural mechanism that renders it insensitive to additional allosteric activation.

- Figure 5A: Structural alignment between an AlphaFold predicted model and a crystal structure of SHIP2 in a closed conformation (top right). The figure does not appear to make sense. The PTP domain appears very similar in both structures, with the only difference being the orientation of the SH2 domain. Did AlphaFold provide alternative structures that closely resemble the closed conformation?

We thank the reviewer for this insightful question. In Figure 5A, the structures were superimposed on the PTP domain because the defining conformational difference between open and closed SHP2 states is the position of the SH2 domains; the PTP domain itself remains largely unchanged. Because Y62D introduces a single substitution within the nSH2 domain, close alignment of the PTP domains between the AlphaFold-predicted and experimentally solved structures is expected and consistent with known SHP2 conformational behavior.

Regarding alternative conformations, AlphaFold2 did generate multiple models for SHP2 Y62D. As shown in the revised Supplementary Figure 5, the top 4 ranked models adopt open conformations, whereas the 5th ranked model more closely resembles the closed state. These outputs are in agreement with our HDX-MS and crystallographic data demonstrating that SHP2 Y62D can sample both conformations but preferentially occupies the open conformation. All relevant AlphaFold models have now been included in Supplementary Figure 5.

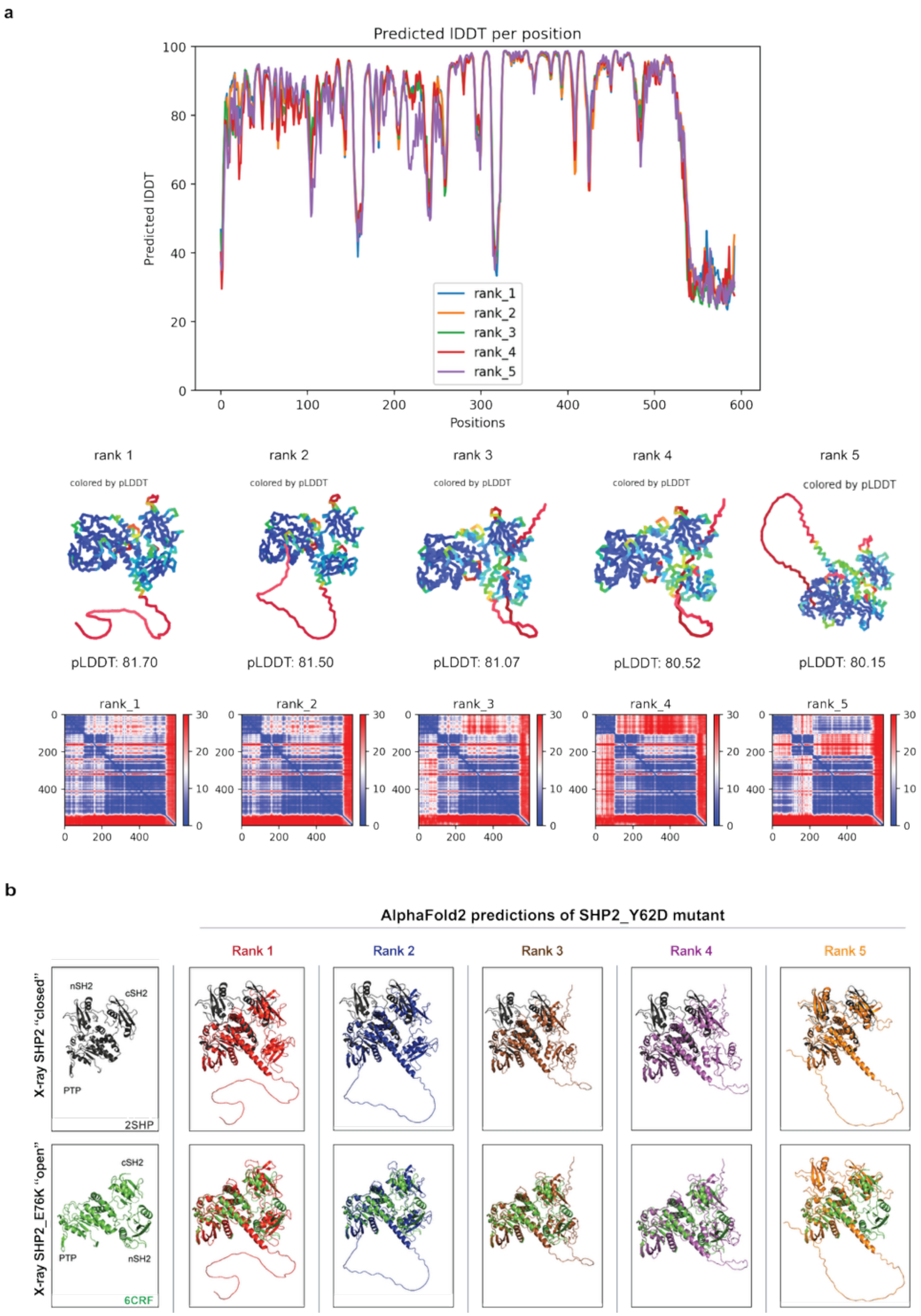

Supplementary Figure 5

- Figure 5C: The percent exchange structure map
  The preparation of the fully-deuterated standard poses an issue. The point of doing a fully-deuterated experiment is to obtain the maximum deuterium uptake of each peptide under the same labeling strength (pH, temperature, and deuterium dilution factor) as that of the labeling experiment. This allows us to normalize the deuterium uptake of each peptide and express them as the percent exchange. This is important since the structural flexibility of different regions in the same state can be compared. For example, the structural flexibility of region A and region B in the same protein (state) can be compared since a fully-deuterated experiment corrects for the back-exchange difference between the two regions (corrects for back-exchange dependence on amino acid composition/sequence of peptides). It also enables the comparison of the percent exchange between different proteins, provided that the fully-deuterated experiments are appropriately performed.

In our HDX/MS workflow, which follows standard practice in the field, the fully deuterated control is used to determine the maximum possible deuterium uptake for each peptide. To obtain this, we fully unfold the protein, deuterate it, and measure the resulting mass of each peptide. These values allow us to correct all forward-exchange data for D-to-H back-exchange, which occurs during chromatographic processing. In our hands, back-exchange is typically 10-40%, and we exclude peptides with excessive back-exchange (>50%) to maintain high confidence.

After this correction, deuterium uptake can be compared within the same peptide across conditions (e.g., WT vs mutant). However, as the reviewer notes, back-exchange correction alone does not make peptides with different amino-acid compositions directly comparable, because each sequence has its own intrinsic exchange rates that cannot be precisely normalized on a residue-by-residue basis. For this reason, percent exchange should not be compared across peptides of different sequences, but comparisons among identical peptides (e.g., WT vs point mutants) are valid and appropriate.

- The labeling strength is highly dependent on labeling temperature. So, the authors' result vastly underestimates structural flexibility due to the higher temperature employed for the full-deuterated experiment. For example, consider the following scenario where you are analyzing a 10-residue peptide. The maximum possible uptake is 9 (no proline), and you want to determine the fully-denatured/deuterated uptake of this peptide. The labeling strength is lower at 15 °C than at 37 °C. So, the experimentally determined maximum uptake of all peptides will be higher at 37 °C (Say Duptake = 7 for this peptide) than at 15 °C (Duptake = 5). If you then determine the same peptide uptake to be 3, the calculated percent exchange at 37 °C and 15 °C is 43% and 60%, respectively. A viable remedy for this issue is to correct the deuterium uptake data using the different temperature factor (or repeat the fully-deuterated experiments).

We thank the reviewer for raising this point and appreciate the opportunity to clarify the purpose of the full-D control in our HDX-MS experiments. We appreciate the reviewer's broader point that labeling conditions influence absolute percent-exchange values. The full-D control serves a distinct purpose from the forward-exchange reaction and is not intended to replicate its

conditions. Its sole purpose is to provide an accurate estimate of back-exchange so that deuterium-uptake curves for each peptide can be properly corrected.

To achieve complete deuteration, the protein must be fully unfolded, which we accomplish by increasing the temperature before quenching. This ensures that all exchangeable amides are maximally exposed and allows us to determine the true upper limit of deuterium incorporation. Because this step is designed to measure back-exchange after quenching, rather than any property of the forward-exchange reaction, the full-D sample does not need to match the temperature or folding state of the time-point samples prior to the quench.

If the full-D control were performed under the same folded-protein conditions as the time-point samples, many residues would remain incompletely exchanged even after 48 hours, leading to an inaccurate estimate of back-exchange. For this reason, it is standard practice to use unfolded conditions for the full-D control to ensure complete deuteration and accurate peptide-level correction.

- Alternatively, since Fig.5C uptake plots (in Da, the same peptide uptake comparisons) are appropriate, I suggest that the authors keep these exchange uptake data. It will be helpful if the authors indicate/highlight peptide regions in the SHP2 structure more clearly.

We agree that the uptake plots in Fig. 5C are appropriate for comparing identical peptides across matched proteins, and we have retained these data in the revised manuscript. To improve clarity, as suggested, we have now indicated the N- and C-terminal positions of the four peptides directly on the SHP2 structural model and have revised Figure 5C accordingly. All peptides analyzed are clearly annotated in the Supplemental Information.

c

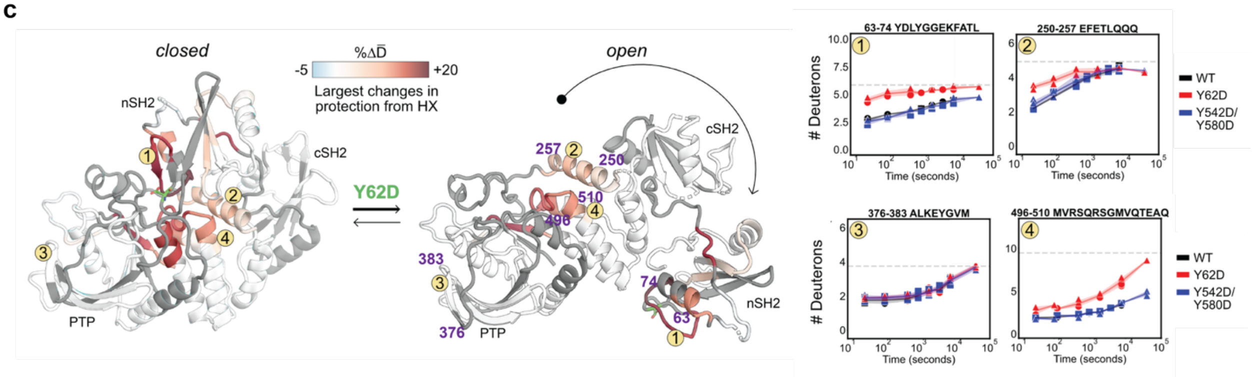

Revised Figure 5c

- Also, based on the HDX-MS results, can the authors speculate how the pY62 mutant (Y62D) confers drug resistance? Is the Y62D mutant drug-resistant like the pY62 mutant?

SHP099 and related allosteric inhibitors act as molecular glues that bind at the nSH2-PTP interface and stabilize the autoinhibited, closed conformation of SHP2. Our HDX-MS data show that the Y62D phosphomimetic shifts the conformational equilibrium toward a more open state, reducing the stability of this autoinhibited interface. This provides a mechanistic basis for

reduced inhibitor sensitivity: because the closed conformation is less populated, the SHP099 binding interface is correspondingly less accessible.

Consistent with this model, the biochemical inhibition assays in Supplementary Figure 7 demonstrate that SHP2 Y62D exhibits decreased sensitivity to SHP099 compared with wild-type SHP2, indicating that the phosphomimetic is drug-resistant at the protein level.

In summary, the title of this manuscript does not align with the content. The manuscript requires major revision, with a substantial number of additional experiments needed to show a connection between pY62 activation and drug resistance.

We have now performed the additional experiments requested throughout the review—including direct biochemical measurements of inhibitor sensitivity for SHP2 Y62D, on-target validation of kinase-inhibitor treatments, and expanded structural and HDX-MS analyses. Together, these new experiments strengthen the mechanistic linkage between pY62 activation and reduced sensitivity to allosteric SHP2 inhibition and bring the title and conclusions of the manuscript into full alignment with the expanded experimental evidence.

Reviewer #2 (Remarks to the Author):

This paper reports a thorough exploration of phosphorylation of SHP2 on Y62 as a molecular mechanism of sustaining SHP2 in an active form and as a mechanism of resistance to SHP2 inhibition. The data generally support the conclusions drawn by the authors. Overall, the work will be a meaningful contribution to the field, and I am supportive of publication after the refinement of the x-ray structure is completed (see below) and the authors make the requisite textual edits to give the prior work proper recognition.

We thank the Reviewer for these favorable comments.

Major points:

- The authors unnecessarily downplay the prior identification of pY62 that was reported in two other manuscripts. They do cite both papers, but their text in the introduction and discussion minimizes the value of the earlier work and makes it appear as if the authors are making a breakthrough discovery of Y62 phosphorylation. They should be more forthright in acknowledging the prior work documenting Y62 phosphorylation as a resistance mechanism. What makes this work stand out is that it more fully explores the generality of Y62 phosphorylation across cancer cell types and the scope of contexts in which the modification may impact signaling, reports a pY62 phosphoselective Ab to probe direct versus indirect induction of the pY mark, thereby assigning phosphorylation to SFKs, rather than the RTKs, and uses Y62D as a phosphomimetic for structural analysis, HX-MS, and biochemical studies to complement the cellular work.

We appreciate the reviewer's point and agree that prior studies made important contributions by identifying SHP2 pY62 in cytokine-stimulated hematopoietic cells and in FLT3-driven AML models with acquired SHP099 resistance. Our intent was not to minimize these findings, and we have revised the Introduction and Discussion to acknowledge this work more directly and prominently.

Specifically, we now state in the Introduction that pY62 was first observed in cytokine-stimulated hematopoietic stem cells (Louria-Hayon, *PNAS* 2013) and that pY62 was later found to be enriched in SHP099 resistant FLT3-driven acute myeloid leukemia cells and was associated with SHP-099 resistance possibly through enforcing an open, active conformation of SHP2 (Pfeiffer, *Cancer Res* 2022). In the Discussion, we explicitly note that pY62 has been linked to SHP2 inhibitor resistance in leukemia and place our work in this context—emphasizing that our study extends these observations by defining the broader prevalence of pY62 across cancer types, demonstrating SFK-mediated phosphorylation using a phosphospecific antibody, and integrating structural, biochemical, and HDX-MS analyses using the Y62D phosphomimetic.

- Review of the x-ray structure table, text, pdb validation report, model, and map shows that the claim in the table of 2.63 Å resolution is not congruent with the text, which states a resolution of 2.9 Å..,

We apologize for this inconsistency. We have corrected the reported resolution in the text from 2.9 Å to 2.63 Å, which now matches the data processing and refinement statistics presented in the table.

- and that the data set is not complete, particularly in the outermost shell.

We agree that the dataset shows reduced completeness in the highest-resolution shell, and this is a direct consequence of the anisotropic diffraction that we observed across all SHP2 crystals collected for this project. We attempted to improve the quality of the crystals through optimization of both the crystallization and cryo-protection conditions, but the quality of the crystals did not improve significantly.

To ensure that all useful reflections were used during refinement the data were processed with STARANISO (Global Phasing). STARANISO applies an anisotropic cutoff limit to include reflections from strongly diffracting regions of reciprocal space and exclude reflections from weakly diffracting regions of reciprocal space. The inclusion of the strong reflections during refinement improves the accuracy of the model and the interpretability of the electron density maps, while the exclusion of the weak reflections reduces the amount of noise in the electron density maps. While the use of an anisotropic cutoff limit results in an apparently reduced data completeness, this is only through the exclusion of reflections that would not improve the accuracy of the model or the quality of the electron density maps.

- The Rwork and Rfree values are unexpectedly high, even for the claimed 2.9 Å resolution, and the quality of the map and model fit could be substantially improved.

We agree that the Rwork and Rfree values are higher than typically expected at this resolution, which is a recognized consequence of refining against an anisotropic dataset. Nevertheless, the electron-density maps are sufficiently interpretable to support the key conclusion we draw from the structure—namely, that SHP2 does *not* adopt an open conformation in this crystal form. Because our structural interpretation relies on the *absence* of features consistent with the open state rather than on modeling fine details of an activated conformation, the quality of the maps is adequate for this purpose.

- Attention to the refinement might also clarify whether there could be a bridging H2O molecule between D61 and D62.

There are two waters in the close vicinity of Asp61 and Asp62 (in chain A, W/117 and W/144). We have included a figure here where the electron density around those two residues is depicted alongside the final coordinates (rmsd 1.0 sigma, the depicted chain is chain A).

From the attached image it becomes clear that the two waters and the carboxy groups of Asp61 and Asp62 form a trapezoid. However, the distances between the waters and the carboxy groups are too high to build a water network by themselves. There are no indications in the electron density that further water molecules are bound close to Asp61 or Asp62.

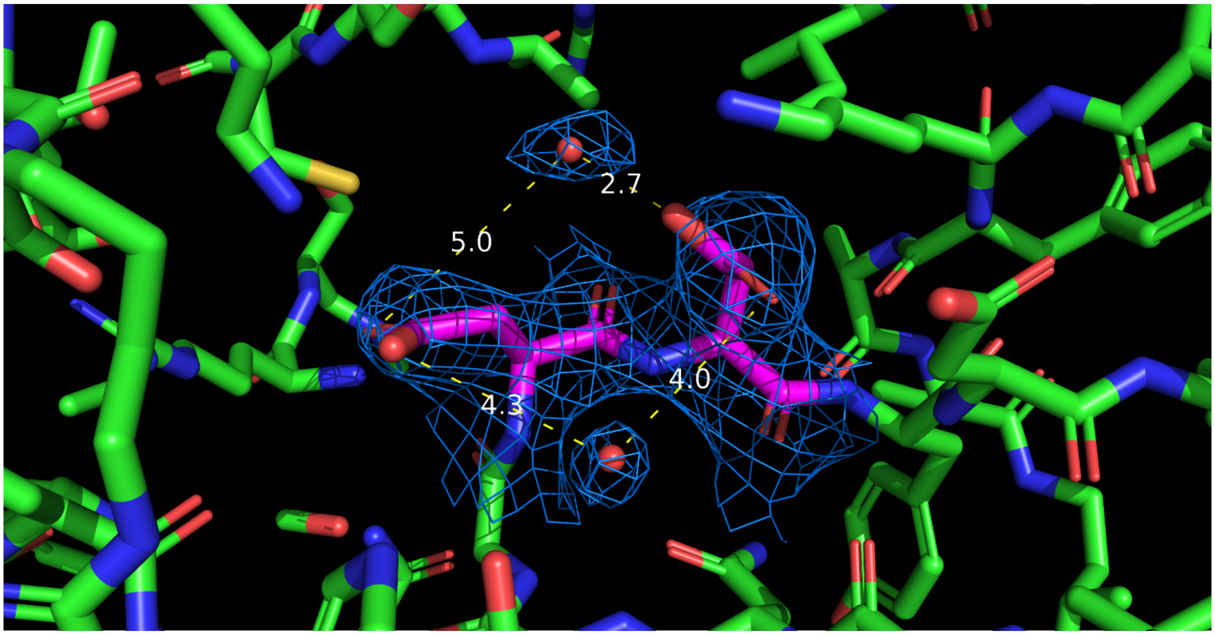

Figure: Electron density of Asp61 and Asp62 (in purple) including surrounding (5 Å) residues. Protein in green, the two water molecules are depicted as red spheres and the distance to Asp61/Asp62 is shown.

- One other minor point - the enzymatic data to measure catalytic parameters appear to substantially overestimate the activity of the WT (unmutated) protein compared to values published elsewhere. The basal activity of wt SHP2 is difficult to measure accurately, but it is not clear why that would be true.

In revisiting our analysis, we realized that the initial activity values had not been normalized using a standard calibration curve, which can lead to overestimation of absolute catalytic rates. We have now reprocessed the data using an appropriate standard curve, and the revised values—shown in Figure 4A—are consistent with published basal activities reported for WT SHP2. We apologize for this error and thank the reviewer for drawing attention to this point.

We agree that accurately determining the basal activity SHP2, which is an intrinsically autoinhibited phosphatase, is inherently challenging particularly at very low substrate turnover. Although additional measurements at lower DiFMUP concentrations could further refine these estimates, the revised kinetic parameters now fall within the expected range and align with previously reported values.

Reviewer #3 (Remarks to the Author):

SHP2 is a phosphatase and a critical mediator of receptor tyrosine kinase (RTK)-driven RAS/mitogen-activated protein kinase (MAPK) signaling. Despite promising preclinical data, SHP2 inhibitors have shown minimal clinical efficacy, with no defined clinical mechanisms of primary resistance. Therefore, it is important to elucidate the underlying mechanism on the lack of clinical response of SHP2 inhibitors in human cancer and to develop new therapeutic strategies to target SHP2.

In this study, the authors elucidated phosphorylation of SHP2 at tyrosine 62 (pY62) as a hotspot phosphorylation site in the proteome and RTK-driven tumor types in patients. They demonstrated that SRC family kinases directly phosphorylate SHP2 at Y62, downstream of but not directly phosphorylated by RTKs. Using biochemical and biophysical analyses, the authors showed that SHP2 Y62D (a phosphor tyrosine mimic) enforces an open, active conformation, resulting in constitutive phosphatase activation that is sufficient to activate MAPK signaling and confer resistance to allosteric SHP2 inhibitors. Their findings establish that SHP2 pY62 is a phosphorylation hotspot phenocopying mutational activation, a mechanism of primary resistance to SHP2 inhibitors, and a cancer drug target distinct from wildtype SHP2.

This study employed a number of complementary approaches and the experiments were well done. The conclusion was solid and well justified. The manuscript was well written and easy to follow. This study answered an urgent and important question for the SHP2 filed and provided novel therapeutic approaches to target active SHP2, instead of inactive SHP2. It is recommended that the study be published in Nature Communications.

We appreciate the reviewer’s comments about our findings.

Reviewer #1 (Remarks to the Author):

I thank the authors for taking considerable time to address my concerns. I would also like to commend them for performing through experiments and for providing a clear and thoughtful rebuttal to my comments.

I would like to offer one additional point regarding HDX-MS analysis. Comparison of percent deuterium exchange across different peptides is appropriate provided that fully deuterated control experiments are performed. In response, the authors state that "back-exchange correction alone does not make peptides with different amino-acid compositions directly comparable,because each sequence has its own intrinsic exchange rates that cannot be precisely normalized on a residue-by-residue basis."

However, the purpose of back-exchange correction is precisely to normalize for sequence-dependent effect, including intrinsic exchange arising from amino acid composition. Indeed, examination of back-exchange values across peptides often reveal clear sequenced dependence – for example, histidine-containing peptides typically show higher back exchange – demonstrating that such correction explicitly account for intrinsic sequence effects. Once normalized using fully deuterated controls, percent deuterium exchange therefore allows meaningful comparison between different peptides.

If percent exchange across peptides were fundamentally invalid, the utility of back-exchange correction would be unclear. In that case, analysis would be restricted to absolute deuterium uptake (Da), limiting comparisons to identical peptides across conditions and substantially reducing the interpretive power of HDX-MS.

Furthermore, full deuteration can be reliably achieved for most soluble proteins within 1-3 minutes at 25°C using high denaturant concentration (e.g., 6 M guanidine-DCl, or 8 M urea-d4), provided that the labeling strength is sufficient (~20-fold deuterium dilution). I recommend the authors to visit: https://hx2.med.upenn.edu/download.html, which calculates theoretical intrinsic exchange as well as exchange rates in various conditions (pH, temp). Although the site only calculates these rates for given peptide sequences, it may be useful to estimate full exchange times.

We thank the reviewer for this additional clarification and agree with the general principle that, when fully deuterated controls are appropriately performed, percent deuterium exchange can be used to compare structural flexibility across different peptides.

In our HDX-MS workflow, fully deuterated controls were performed for all peptides and used to correct for D-to-H back-exchange, with peptides exhibiting excessive back-exchange excluded. This enables robust peptide-level normalization and supports valid comparison of deuterium uptake under matched conditions.

In the context of this study, we intentionally restrict interpretation to comparisons of identical peptide sequences across matched protein variants (WT versus point mutants), providing a conservative and direct assessment of mutation-induced conformational changes.

To make this analytical choice explicit, we have added the following sentence to the Methods section: "Fully deuterated controls were used for peptide-level back-exchange correction; although percent-exchange comparisons across different peptides are feasible in principle, all analyses here are confined to comparisons of identical peptide sequences across matched protein variants."

We believe this clarification addresses the reviewer's point while accurately reflecting both standard HDX-MS practice and the analytical framework used in this work.

Reviewer #2 (Remarks to the Author):

The authors adequately addressed my comments about the referencing of prior work, their X-ray data, and the catalytic activity of the autoinhibited WT enzyme. I defer to the first reviewer regarding comments about SFKs as the kinases responsible for Y62 phosphorylation, as well as their other concerns.

We appreciate the reviewer's comments.
